# Phosphoinositide-3-Kinase Is the Primary Mediator of Phosphoinositide-Dependent Inhibition in Mammalian Olfactory Receptor Neurons

**DOI:** 10.3389/fncel.2016.00097

**Published:** 2016-04-11

**Authors:** Kirill Ukhanov, Elizabeth Corey, Barry W. Ache

**Affiliations:** ^1^Department of Pharmacology and Therapeutics, University of FloridaGainesville, FL, USA; ^2^Whitney Laboratory, Center for Smell and Taste, McKnight Brain InstituteGainesville, FL, USA; ^3^Department of Biology and Neuroscience, University of FloridaGainesville, FL, USA

**Keywords:** odor inhibition, olfactory receptor neurons, phosphoinositide signaling, *en face in vivo* imaging, single cell PCR

## Abstract

Odorants inhibit as well as excite primary olfactory receptor neurons (ORNs) in many animal species. Growing evidence suggests that inhibition of mammalian ORNs is mediated by phosphoinositide (PI) signaling through activation of phosphoinositide 3-kinase (PI3K), and that canonical adenylyl cyclase III signaling and PI3K signaling interact to provide the basis for ligand-induced selective signaling. As PI3K is known to act in concert with phospholipase C (PLC) in some cellular systems, the question arises as to whether they work together to mediate inhibitory transduction in mammalian ORNs. The present study is designed to test this hypothesis. While we establish that multiple PLC isoforms are expressed in the transduction zone of rat ORNs, that odorants can activate PLC in ORNs *in situ*, and that pharmacological blockade of PLC enhances the excitatory response to an odorant mixture in some ORNs in conjunction with PI3K blockade, we find that by itself PLC does not account for an inhibitory response. We conclude that PLC does not make a measurable independent contribution to odor-evoked inhibition, and that PI3K is the primary mediator of PI-dependent inhibition in mammalian ORNs.

## Introduction

The process of odor detection and identification begins when odorants bind to olfactory receptors (ORs) expressed in the cilia of primary olfactory receptor neurons (ORNs) in the olfactory epithelium (OE) to activate signal transduction. Odorants can inhibit as well as excite ORNs thereby integrating their responses to complex odor mixtures (Ache, 2010; Thomas-Danguin et al., 2014; Schubert et al., 2015; Corey and Ache, 2016). The canonical excitatory signaling pathway in mammals begins with odorant-evoked activation of adenylyl cyclase III (ACIII) through the olfactory G protein Gα_olf_, resulting in an increase in the second messenger cAMP. Subsequent opening of cyclic nucleotide-gated (CNG) channels and Ca^2+^-activated Cl^-^ channels depolarizes the ORNs, which fire action potentials to transmit the signal to the olfactory central nervous system (CNS). In contrast, much less is known about the mechanisms through which odorants decrease the output of ORNs, a process referred to as odor-evoked inhibition.

Odor-evoked inhibition at the level of ORN is often associated with competitive interaction between the cognate ligand and an antagonist, as was studied in detail with the rat I7 receptor (Peterlin et al., 2008). However, there is growing evidence that at least one other type of odor-evoked inhibition is mediated by phosphoinositide (PI) signaling through activation of phosphoinositide 3-kinase (PI3K; Spehr et al., 2002; Ukhanov et al., 2011a,b, 2013) and that activation of the cyclic nucleotide-based excitatory and PI3K-based inhibitory signaling pathways in a ligand biased manner provides the basis for Ligand-induced Selective Signaling (LiSS; e.g., (Kenakin, 2003; Park, 2012; Shukla et al., 2014) in mammalian ORNs. As phospholipase C (PLC) and PI3K can be activated in concert in other cellular systems to regulate cell motility and chemotaxis (Kölsch et al., 2008), question arises as to whether PLC is also part of the PI pathway mediating inhibitory transduction.

This possibility gets traction from the finding that in some mammalian ORNs relief of odor-evoked inhibition appeared to require pharmacological blockade of both arms of the PI signaling pathway, i.e., PI3K and PLC, not just PI3K (Spehr et al., 2002). There is also evidence that odorants can activate PLC as well as PI3K in olfactory ciliary membranes (Vogl et al., 2000; Klasen et al., 2010), and isoforms of both enzymes have been detected at the level of the OE (Bruch et al., 1995; Brunert et al., 2010; Ukhanov et al., 2010; Szebenyi et al., 2014), in some cases in the olfactory cilia (Brunert et al., 2010; Ukhanov et al., 2010). These findings raise the possibility that PLC and PI3K both contribute to an inhibitory signaling branch of LiSS.

We now establish that multiple PLC isoforms are expressed in the transduction zone of rat ORNs, odorants can activate PLC in ORNs *in situ*, and pharmacological blockade of PLC enhances the excitatory response to an odor mixture in some ORNs in conjunction with PI3K blockade. However, we find that PLC alone does not account for an inhibitory response. We conclude that PLC by itself does not make a measurable contribution to odor-evoked inhibition, and that PI3K is the primary mediator of PI-dependent inhibition in mammalian ORNs.

## Materials and Methods

Adult female Sprague-Dawley rats were used in experiments. All procedures were carried out in accordance with protocols approved by the University of Florida IACUC. All experiments were performed at room temperature (22–25°C).

### Calcium Imaging of Dissociated ORNs

Experiments were performed as described previously (Ukhanov et al., 2011b). In brief, olfactory tissue was dissected in ice-cold modified artificial cerebrospinal fluid (ACSF) saturated with 95% O_2_ and 5% CO_2_ that contained (in mM): 120 NaCl, 25 NaHCO_3_, 5 KCl, 1.25 Na_2_HPO_4_, 1 MgSO_4_, 1 CaCl_2_, 10 glucose, 305 mOsm, pH7.4. After enzymatic digestion in papain (1 mg/mL) supplemented low-Ca (0.6 μM buffered with CaEGTA) ACSF at 37°C for 15 min, the tissue was gently washed with normal oxygenated ACSF and accurately triturated with a large bore fire polished glass transfer pipette. The resulting suspension was filtered through a 40 μm cell strainer (Fisher Scientific) and stored at 4°C with added TurboDNAse (1:100 dilution, Ambion) to prevent cell clumping. An aliquot of the suspension was mixed with 10 μM Fluo-3/AM (AnaSpec) containing 0.04% Pluronic F127 and placed on a glass coverslip coated with 1 mg/mL concanavalin A (Sigma-Aldrich) in a recording chamber (RC22, Warner Instruments). The chamber was transferred to the stage of an inverted microscope (Axiovert 200, Zeiss) equipped with a 10× /0.5NA Fluar objective. To increase the sample size, in each experimental session the field of view was further expanded using a 0.63× reducer video tube (Diagnostic Instruments) between the microscope and the camera. Depending on the quality of the preparation, approximately 300 ORNs sensitive to IBMX/forskolin stimulation could be analyzed at once. The cells were illuminated at 500 nm (BP 500/20 nm, Omega Optical, USA) and the emitted light was collected at 530 nm (BP 530/20 nm, Omega Optical, USA) by a 12-bit cooled CCD camera (ORCA R2, Hamamatsu, Japan). Both the illumination system (Lambda DG-4, Sutter Instruments, CA, USA) and image acquisition were controlled by Imaging Workbench six Software (INDEC BioSystems).

Each cell was assigned a region of interest (ROI) and changes in fluorescence intensity within each ROI were analyzed and expressed as the peak fractional change in fluorescent light intensity F/F0 where F0 is the baseline fluorescence before odor application. Responses were detected and measured when the change in fluorescence intensity exceeded two standard deviations above the median noise level. For quantitative comparison, the peak amplitudes of the responses of different cells were normalized to the saturated responses elicited by application of a mixture of 100 μM IBMX (a phosphodiesterase inhibitor) and 10 μM forskolin (a selective agonist of adenylate cyclases) to robustly activate the cAMP signaling pathway. Application of IBMX/forskolin was also used to identify functional ORNs.

### *En Face* Imaging of the OE Ectopically Expressing PIP_2_ Probe and GCaMP6f Calcium Probe

Plasmids encoding the adenoviral backbone genes and the shuttle vector were provided by Dr.Jeffrey Martens (University of Florida) and viruses were prepared according to established protocols (McIntyre et al., 2012). Briefly, for ectopic expression in native tissue, PLCdelta1-PH:GFP and GCaMP6f were cloned into the adenoviral vector pAd/V5/dest and virus was propagated in HEK293A cells. Adenoviral particles were isolated with the Virapur Adenovirus mini purification Virakit (Virapur, San Diego, CA, USA) and dialyzed in 2.5% glycerol, 25 mM NaCl and 20 mM Tris-HCl, pH 8.0 at 4°C before storage at −80°C. Rats were anesthetized with a Ketamine/Xylazine mixture and 10–15 μL of the viral solution was delivered intranasally as a single injection per nostril. Animals were used for experiments at 7–14 days post-infection. Entire turbinates and septums were dissected and kept on ice in a Petri dish filled with oxygenated ACSF. For imaging a small piece of the OE was mounted in the perfusion chamber with the apical surface facing up. The chamber was transferred to the stage of an upright microscope Axioskop2F equipped with a 40× NA 0.75 water-immersion objective lens. Experimental solutions were applied directly to the field of view through a 100 μm diameter needle made of fused silica and connected to the 9-channel Teflon manifold (Biologic, France). Each perfusion channel was controlled by electronic valves (VC-6, Warner Instruments).

To observe translocation of the PIP_2_ specific probe PLCdelta1-PH:GFP, individual dendritic knobs were imaged through an additional 2.5× video magnifying converter (Zeiss). The same optical configuration and illumination used to image dissociated cells was utilized for *en face* imaging of OE ectopically expressing PLCdelta1-PH:GFP or the calcium sensor GCaMP6f. Translocation was measured as an increase in global intra-knob cytoplasmic fluorescence upon application of the odor and presented as (F-Fo)/Fo ratio. The calcium response presented as an increase of GCaMP6f fluorescence emanating from the knob and underlying cell body. A standard eGFP filter cube BP490 nm/535 nm (Omega, Brattelboro) was used to image both probes. Acquisition and illumination were controlled as described above.

High resolution *en face* imaging of freshly dissected OE was performed on an inverted confocal microscope Nikon Ti/A1R+ controlled by the NIS Elements 4.20 Software. Images were processed using ImageJ (NIH^1^) and assembled in CorelDraw13 (Corel).

### Assay of PI3K Activity

A constitutively active form of the catalytic subunit p110gamma of PI3K fused to a membrane anchor motif CAAX, (PI3Kgamma-CAAX; provided by Dr.Wyman, University of Fribourg, Switzerland) was co-expressed with the PIP_3_ probe Btk-PH:GFP (provided by Dr.Balla, NIH/NICHD) in HEK293T cells. PI3K activation results in an accumulation of PIP_3_ and in turn recruitment of Btk-PH:GFP to the plasma membrane. Application of the PI3K inhibitor LY294002 reduces the PIP_3_ level in the membrane and Btk-PH:GFP translocates to the cytoplasm. PI3K-dependent changes in fluorescence were measured using the same optical configuration and illumination used to image dissociated ORNs and the PIP_2_ probe.

### Drugs, Odorants, and Solution Application

3-isobutyl-1-methylxanthine (IBMX) and 7β-acetoxy-8,13-epoxy-1α, 6β, 9α-trihydroxylabd-14-en-11-one (forskolin) were obtained from Sigma-Aldrich. LY294002 (Tocris), U73122, U73343 and edelfosine (Cayman). Reagents were dissolved in anhydrous DMSO and the stocks were kept at −20°C. Odorants were delivered as aqueous solutions prepared in freshly oxygenated ACSF. Octanol (OOL) and citral (CIT) were of the highest purity (Fisher Scientific) and were prepared as 0.5 M stock solutions in anhydrous DMSO. A complex odor mixture (Henkel-100, provided by Prof.Hatt, University of Bochum, Germany) was diluted 1:10 in DMSO and then mixed to the final reported dilution with ACSF. ACSF supplemented with 0.1% DMSO, the odorant carrier, served as the control solution. Odorant stocks were kept at −20°C and the final aqueous solutions were prepared on the day of the experiment.

### Data Analysis

All data are expressed as mean ± SEM. All analyses were performed with pClampfit 10.2 (Molecular Devices), Microsoft Excel and SigmaPlot 10 (Systat Software, CA, USA) Software.

### RT-PCR

Odor-responsive dissociated ORNs identified by calcium imaging were collected directly into RT-PCR buffer and immediately frozen. Thawed cells were used for reverse transcription with an anchored oligo dT primer using a Verso cDNA kit (Thermo Fisher). To allow analysis of the expression of a number of genes, the cDNAs were pre-amplified with Primestar HS DNA polymerase (Takara) using a cocktail of gene-specific primers and a primer nested within the anchored-dT primer by incubating at 95°C for 2 min followed by 15 cycles of 95°C for 30 s, 50°C for 30 s 72°C for 1 min, then 72°C for 5 min. Samples were aliquoted and immediately frozen for later analysis. Beta actin primers were used to test for contamination with genomic DNA prior to analysis of PLC expression and olfactory marker protein (OMP) primers were used to confirm that the cells were canonical ORNs. PCR detection of the PLC isoforms was performed with gene specific primers (*PLCG2f* 5′ACTCTCTTCTCTCTCAGCCTCCG3′, *PLCG2r* 5′CAACAAATTCAAGACGACGGTTGTG3′, *PLCE1f* 5′TCCAAGTGCACGATGTCTCTCCAG3′, *PLCE1r* 5′GACTGGTAGCCCGCTGTGTCAC3′, *PLCB3f* 5′CCAGAACCGACAAGTACAGAGCC3′, *PLCB3r* 5′TCCCAGCTGCATGACCATTGC3′) and 10 μl of each reaction was analyzed by agarose gel electrophoresis.

### Whole Mount Immunohistochemistry

Standard immunofluorescent staining of cryostat sections of fixed OE was not of sufficient quality to resolve anti-PLC labeling. Instead, we used whole mount fixation of the OE and *en face* imaging. To further enhance the labeling, fixed tissue was incubated in 1% SDS/PBS for 15 min at RT to retrieve antigens (Brown et al., 1996) followed by biotin/streptavidin amplification. Freshly dissected rat noses were fixed for 1–2 h on ice in 4% paraformaldehyde in PBS and then blocked with PBS containing 10% normal horse serum and 0.3% Triton X-100 for 1 h at RT. After washing in PBS, the specimens were incubated with Streptavidin/Biotin blocking solution as per the manufacturer’s instruction (Vector Laboratories) followed by overnight incubation at 4°C with primary antibodies diluted (1:250) in PBS with 0.1% Triton X-100 and with no added serum. Primary antibodies against the respective PLC isoforms were from Santa Cruz Biotechnology. Biotinylated horse anti-rabbit antibody was diluted 1:500 in 1% BSA and incubated with the specimens for 1 h at RT. After extensive washing in PBS, the specimens were incubated with Streptavidin-Dylight-488 conjugate (1:200–1:33) in PBS. For double labeling, mouse antibody against acetylated alpha-tubulin (1:1000, Sigma Aldrich) was mixed directly with primary antibody. Secondary anti-mouse Alexa 568 (1:1000) also was added directly to Streptavidin reaction mix. The processed specimens were analyzed using an inverted confocal microscope Leica SP5 (Leica Microsystems). Images were post-processed using ImageJ (NIH^2^) and assembled in CorelDraw13 (Corel).

## Results

### Expression of Multiple Isoforms of PLC at the Single Cell Level in Rat ORNs

**Figure 1:**
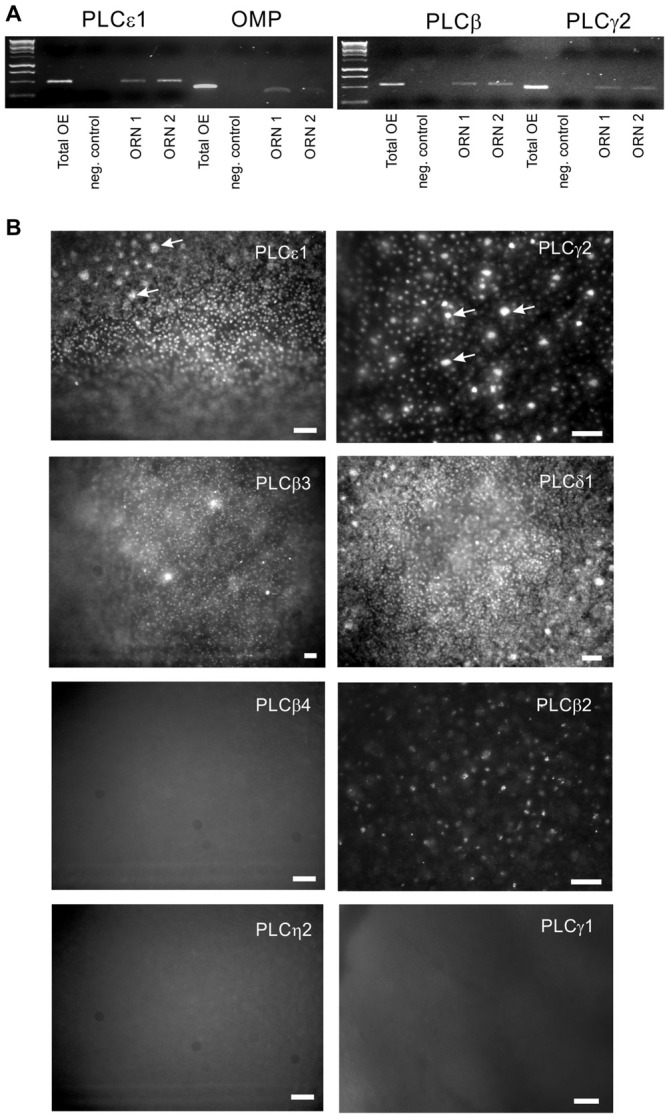
**Multiple isoforms of phospholipase C (PLC) are expressed in olfactory receptor neurons (ORNs). (A)** Single-cell RT-PCR with primers specific for PLCbeta3, gamma2 and epsilon1 was performed on individual odor-responsive rat ORNs. Representative PCRs for two ORNs (ORN1 and ORN2) are shown. Olfactory marker protein (OMP) expression confirms that the cells collected were mature canonical ORNs. Total rat OE cDNA (total OE) was used as a positive control. Both water (negative control) and no RT RNA samples were tested as negative controls. **(B)** Wide-field *en face* immunofluorescent images of whole mount fixed rat OE. In addition to PLCepsilon1, gamma2 and beta3, we have detected the presence of the delta1 isoform in the knobs of ORNs which are visible as a numerous fluorescent dots. Large bright spots represent the apical tips of microvillar cells (arrows). No specific labeling was found with antibodies against the beta4, eta2 and gamma1 isoforms. Scale bars represent 50 μm.

**Figure 2:**
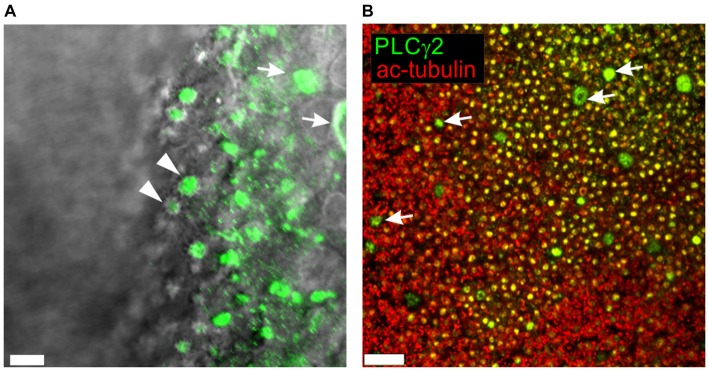
**Higher resolution confocal *en face* images of PLC gamma2 (green) visualized in whole mount rat OE (A) and superimposed on the wide-field DIC image to accentuate localization at the dendritic knobs of ORNs (arrowheads).** Apices and cell bodies of neighboring non-sensory microvillar cells appear brightly labeled (arrows). (B) Acetylated α-tubulin (ac-tubulin; red), a known olfactory ciliary marker protein, was used to counterstain OE labeled with PLCgamma2 (green). Numerous yellow dots are dendritic knobs expressing both PLCgamma2 and ac-tubulin. Knobs are decorated with ciliary proximal segments labeled with ac-tubulin. The apical endings of microvillar cells appear as much larger doughnut like structures lacking any surrounding ac-tubulin labeling (arrows). Scale bars represent 5 μm **(A)** and 40 μm **(B)**.

In the founding experiments leading to this line of investigation (Spehr et al., 2002), a few ORNs responded to an odor mixture only when PI3K and PLC were blocked together, suggesting that the enzymes may act in concert as part of an inhibitory signaling pathway. If PLC contributes to odor-evoked inhibition in mammalian ORNs, at least one isoform of PLC should be present in the transduction compartment (the olfactory cilia). mRNA encoding several isoforms of PLC can be found in the mouse OE (Szebenyi et al., 2014). We confirmed that multiple isoforms could be detected in the whole rat OE by RT-PCR (not shown) and then used calcium imaging to identify individual ORNs that responded to stimulation with an odor mixture (Henkel 100, H-100; 1:10^4^ dilution) for a single cell RT-PCR analysis (Figure 1A). All of the ORNs tested were positive for expression of OMP, proving they were a mature canonical ORNs. All analyzed ORNs expressed both PLCgamma2 and epsilon1, and most of them expressed PLCbeta3 (Figure 1A). Immunofluorescent staining showed that PLCbeta3, delta1, gamma2, and eta1 can be localized to the dendrites of ORNs (Figure 1B). PLCbeta2 was most prominent in the tips of the microvillar cells as previously described (Elsaesser et al., 2005), and was not present in the dendrites of ORNs. Immunofluorescent staining for three other isoforms, beta4, eta2 and gamma1, was negative and served as a negative control (Figure 1B). Higher resolution confocal microscopy supported the localization of PLCgamma2 in the dendritic knobs at the level of proximal ciliary segment as visualized with acetylated tubulin labeling (Figure 2). PLC beta3 and delta1 were similarly localized to the dendritic knobs (not shown), suggesting that several PLC isoforms are present at least at the level of proximal cilia counterstained with acetylated tubulin (Figure 2).

### Odors Can Activate PLC in ORNs *In Situ*

**Figure 3:**
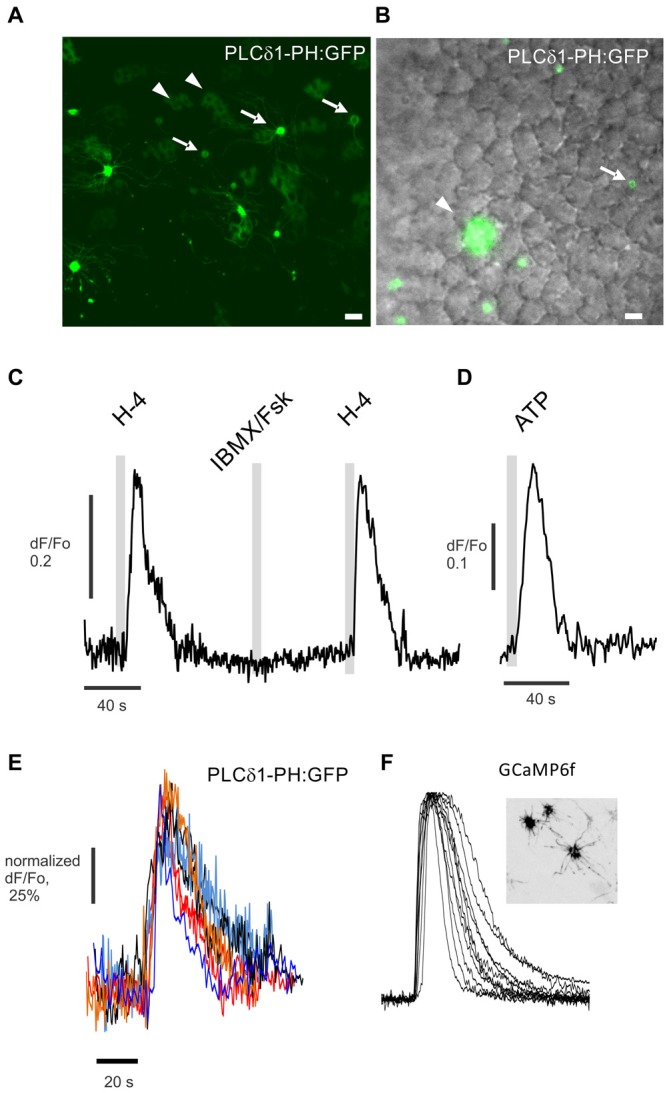
**Odor-evoked activation of PLC detected in the dendritic knobs of ORNs *in situ*. (A)** *En face* confocal imaging of live rat OE expressing the PIP_2_-specific probe PLCdelta1-PH:GFP induced by adenoviral vector. Dendritic knobs bearing multiple cilia (arrows) are present in the field of view along with multiple sustentacular cells (arrowheads). **(B)** Experiment performed with epi-fluorescent illumination as shown. Cells expressing PLCdelta1-PH:GFP are shown in green and superimposed on the DIC image. Scale bars represent 10 μm. **(C)** Application for 5 s of the odor mix Henkel-100 (H-4, 1:10^4^ dilution) but not of IBMX/forskolin (IBMX/Fsk) consistently evokes a robust increase of fluorescence in the cytoplasm of the knob denoted with arrow in **(B)**. **(D)** Application of ATP (100 μM) induced robust PLC activity and translocation of the PIP_2_ probe in sustentacular cells (arrowhead in **B**). **(E)** Normalized traces show odor-evoked PIP_2_ probe translocation in six different dendritic knobs. **(F)** A different preparation from a rat OE expressing the calcium sensor GCaMP6f (insert) used to measure odor-evoked changes in cytoplasmic Ca^2+^ in dendritic knobs. Similarly normalized representative traces of cytoplasmic Ca^2+^ changes measured in 12 knobs of different GCaMP6f expressing ORNs stimulated by application for 5 s of Henkel-100 (H-4, 1:10^4^ dilution). Insert shows *en face* confocal image of an OE with three GCaMP6f expressing ORNs.

If PLC contributes to odor-evoked inhibition in mammalian ORNs, activation by odorants should be detectable. Earlier, we found that odors can activate PLC in ciliary enriched membranes *in vitro*, as well as in ORNs, but not rapidly (Klasen et al., 2010). Here we used the same PIP_2_-specific translocation probe PLCdelta1-PH:GFP and virally expressed it in rat OE (Figures 3A,B). H-100 (1:10^4^ dilution) applied for 5 s evoked a quick increase in fluorescence in some ORN knobs, directly reflecting translocation of the probe from the plasma membrane into the cytoplasm of the knobs of 12% of all tested ORNs (Figure 3C, total 92 knobs used). As would be expected, the PLCdelta1-PH probe also translocated to the cytoplasm of sustentacular cells in response to ATP (100 μM), serving as a positive control for the function of the probe (Figure 3D). Notably, translocation of PLCdelta1-PH did not occur in response to activation of the canonical cyclic AMP signaling pathway following application of an IBMX (a phosphodiesterase inhibitor)/forskolin (a adenylyl cyclase activator) mixture (Figure 3C). The kinetics of PLCdelta1-PH translocation were very similar to those of odor-evoked excitation as measured with a calcium sensor GCaMP6f that was virally expressed in a different population of ORNs (Figures 3E,F). The time to peak was 7.72 ± 1.60 s for PLCdelta1-PH:GFP compared to 6.28 ± 0.63 s for GCaMP6f, while recovery back to the baseline occurred in 42.94 ± 5.19 s (PLCdelta1-PH:GFP) and 35.95 ± 1.06 s (GCaMP6f). We conclude that PLC can be activated in ORNs in an odor-dependent manner with kinetics similar to cAMP-dependent signaling, but through a cAMP-independent pathway.

### U73122 Specifically Blocks PLC Without Altering the Activation of PI3K

**Figure 4:**
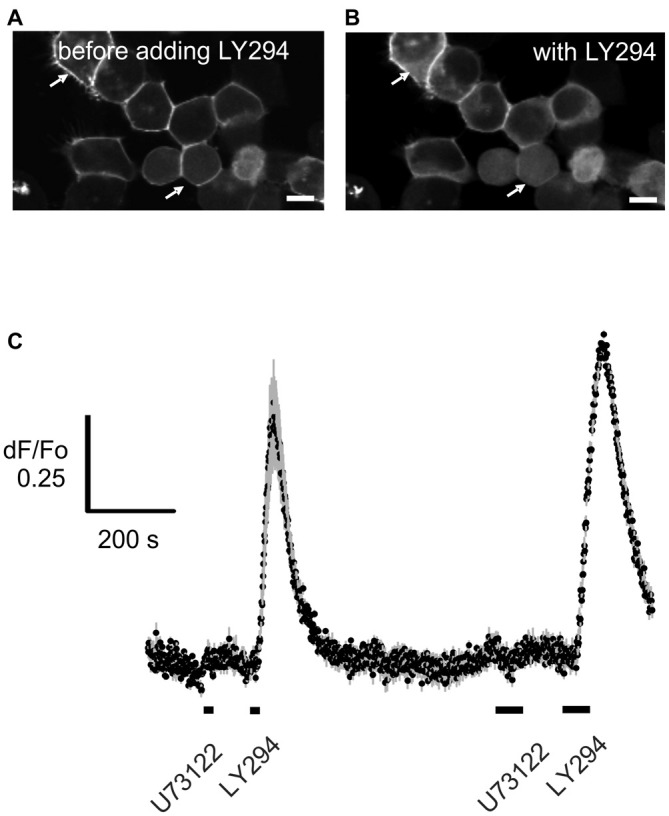
**U73122 does not inhibit the activation of Phosphoinositide 3-Kinase (PI3K).** PI3Kgamma-CAAX, a constitutively active catalytic subunit of PI3K was heterologously expressed in HEK293T cells. Btk-PH:GFP was used as a probe to visualize PIP_3_ generated by the PI3K. **(A)** The probe is localized primarily at the plasma membrane (arrows) whereby **(B)** blocking PI3K with 10 μM LY294002 (LY294) induces translocation of the probe into the cytoplasm. Scale bars represent 10 μm. **(C)** Time course of the translocation induced by application for 10 s (short bar under the trace) and 30 s (longer bar) of LY294002 (LY294) but not by the PLC inhibitor U73122 (10 μM). Data represent the averaged recordings from 25 cells with error bars shown in gray. The data is representative of four independent experiments.

**Figure 5:**
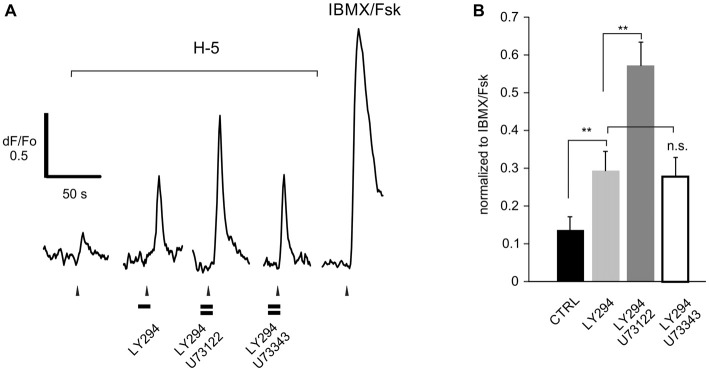
**U73122 is a specific blocker of PLC activity in rat ORNs. (A)** Representative recording from a dissociated ORN that responded to H-100 (H-5, 1:10^5^ dilution) showing that the increased response resulting from PI3K blockade with LY294002 (LY294) is further enhanced by pre-incubation with the active compound U73122. The inactive analog U73343 had no effect. **(B)** Responses were normalized to the response activated by a mixture of IBMX and forskolin (IBMX/Fsk, 100/10 μM each), confirming that these cells are canonical ORNs. A total of 6 cells were used. Asterisks denote significant difference between adjacent data sets (**p < 0.01).

It is important that any pharmacological probe used to implicate PLC acts specifically and in particular, does not cause additional blockade of PI3K. While LY294002 specifically blocks PI3K at the concentrations tested here (Vlahos et al., 1994), the specificity and mechanism of action of U73122 have been disputed (Horowitz et al., 2005; Huang et al., 2013). The finding that edelfosine, a structurally dissimilar PLC blocker, also enhances the effect of PI3K in ORNs provides support for the specificity of U73122, but edelfosine itself can block PI3K (Ruiter et al., 2003). To substantiate that U73122 selectively blocks PLC signaling, we transiently expressed a constitutively active isoform of PI3K, p110gamma-CAAX, (Brock et al., 2003; Kurig et al., 2009) in HEK293T cells and assessed translocation of the probe Btk-PH:GFP as a measure of PIP_3_ production (Balla and Várnai, 2009). In untreated HEK29T cells expressing p110gamma-CAAX, Btk-PH:GFP is predominantly localized to the plasma membrane (Figure 4A). However after incubation with the PI3K blocker LY294002, the probe translocates to the cytoplasm (Figure 4B). In contrast, pre-incubation with the PLC blocker U73122 did not have an effect and did not alter the effect of adding LY294002 (Figure 4C). These results indicate that the concentration of U73122 used here acts specifically on PLC. To “translate” these findings to native ORNs we compared the action of U73122 with its inactive analog U73343 in six ORNs. All six cells showed a PLC-dependent “add on” as described in the following section when treated with U73122 but not when treated with U73343 (Figure 5), suggesting the drug was also acting specifically in native ORNs.

### Pharmacological Analysis of the Potential Contribution of PLC to the Response of ORNs to an Odorant Mixture

**Figure 6:**
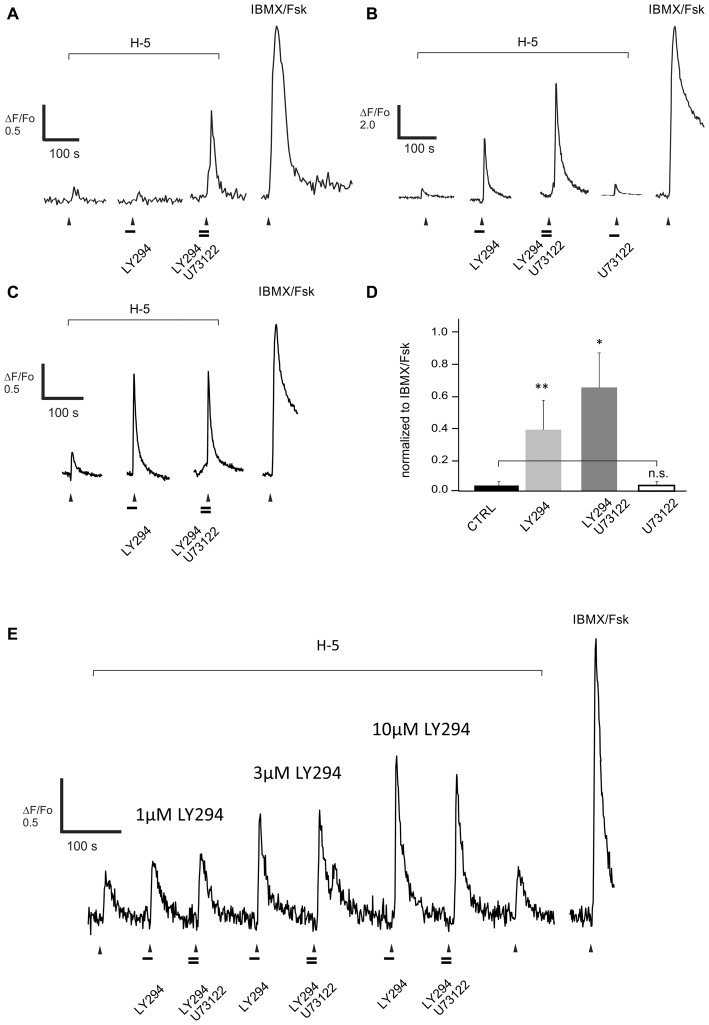
**Blocking PLC activity with U73122 (10 μM) further increases the response evoked by a complex odor mix Henkel-100 (H-5, 1:10^5^ dilution) and enhanced by pre-treatment with the PI3K inhibitor LY294002 (10 μM, LY294). (A–C)** Representative recordings from dissociated rat ORNs clearly show three types of PLC-blocker induced enhancement.** (A)** In one set of cells (5/15) blocking PLC and PI3K in combination was necessary to increase the response to H-100. **(B)** In another set of cells (6/15) blocking PI3K alone enhanced the response and adding U73122 led to a further increase, and **(C)** finally in the third set of cells (6/15) the PLC blocker did not further increase the PI3K-blocker dependent effect. Odor was applied for 5 s at the time indicated with the arrowhead. Horizontal bars indicate 15 s application of the drugs 10 μM LY294002 and 10 μM U73122. **(D)** The net effect of blocking PLC activity in ORNs (*n* = 15) was an increased odor response after blocking activity of PI3K and PLC together. However, in four cells showing an increased response due to PI3K and PLC blockade, pre-incubation with U73122 alone did not induce any effect (empty bar). The response amplitude of each cell was normalized to the maximum evoked by IBMX/Forskolin (100/10 μM, IBMX/Fsk). Asterisks denote a significant difference between data sets (n.s., non-significant; *p < 0.05; **p < 0.01). **(E)** Representative recording from one of two cells showing a PI3K-dependent increase in the response to H-100 (H-5, 1:10^5^ dilution). Reducing PI3K blockade by using lower concentrations of LY294022 (LY294), 1 μM and 3 μM, did not reveal a PLC dependent add-on when 10 μM U73122 was co-applied.

We next asked if we could extend the results of the original study to a larger group of dissociated rat ORNs. The calcium signal evoked by H-100 (1:10^5^) was measured before and after pre-treatment with a PI3K-specific blocker (LY294002, 10 μM) and/or a PLC-specific blocker (U73122, 10 μM), and any change in the amplitude of the signal was measured. The basic logic behind these and the subsequent physiological experiments with native ORNs is that complex odors evoke a net response that reflects the excitation evoked by one or more components tempered by any inhibition evoked by another component of the mixture, and that pharmacologically blocking the inhibition, if any, results in a net increase in the response magnitude. In cases of extreme inhibition where there is no initial detectable excitatory response, this can result in a measurable response after blockade. For quantitative comparison, the peak amplitude of the responses of different ORNs were normalized to the saturated response elicited by application of a mixture of 100 μM IBMX and 10 μM forskolin (Figure 6). Approximately 15% (*n* = 17) of the ORNs that were responsive to an odor mixture (H-100) increased their response following blockade of PI3K. Five of the 17 ORNs responded significantly to the mixture only when both enzymes were blocked, as in the original study (Figure 6A). Combined PI3K and PLC blockade increased the response on average 4.4-fold from 0.07 ± 0.03 to 0.29 ± 0.10. The other twelve cells increased their response to the odor mixture on blocking PI3K alone, but half of them (6/12) further increased their response when PI3K and PLC were blocked together (Figure 6B). PI3K blockade increased the response on average 11-fold from 0.04 ± 0.03 to 0.47 ± 0.22, while co-application of the PLC blocker further increased the response on average 1.7-fold from 0.47 ± 0.22 to 0.79 ± 0.26. This “add-on” effect of blocking PLC, which was not observed in the original study, could suggest the two enzymes work in a graded manner depending on the extent of inhibition evoked by the odor mixture for that particular ORN.

The other half of the ORNs (6/12) that increased their response to the odor mixture on blocking PI3K alone, however, showed no additional effect of blocking PLC (Figure 6C). In these ORNs, blocking PI3K alone increased the response by 3.4-fold from 0.10 ± 0.04 to 0.34 ± 0.09. These cells potentially had near saturated responses following PI3K blockade and therefore may have been unable to generate a measurable increase in output with PLC blockade. The critical metric, however, is whether a particular ORN has more inhibition to be relieved, not the strength of the response *per se*. As a more rigorous assessment, we tested two additional ORNs that showed no additional effect of blocking PLC with several concentrations of the PI3K blocker to determine whether there was an effect of PLC blockade under non-saturating conditions. In both of the cells tested, there was no effect of blocking PLC even at the lowest concentration of PI3K blocker tested when there was clearly more inhibition that could be relieved (Figure 6E).

**Figure 7:**
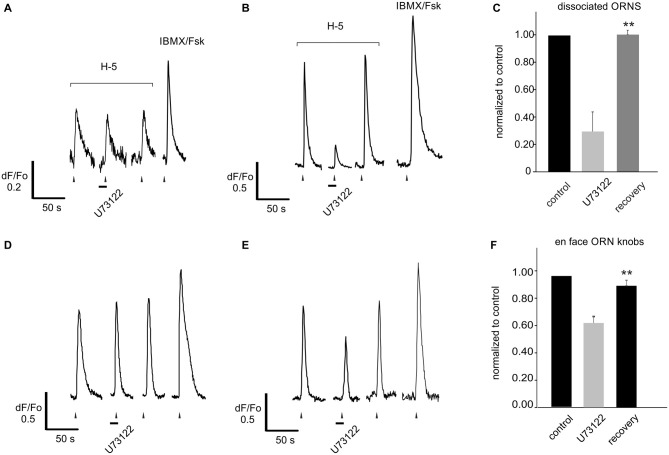
**Blocking PLC alone reveals no increase of the response to a complex odor mix. (A)** Dissociated rat ORNs responding to application of Henkel-100 (H-5, 1:10^5^ dilution) in 29 of 35 cells showed no change when pre-treated with the PLC blocker U73122 (10 μM). **(B)** However in six of 35 cells blocking PLC significantly and reversibly *reduced* the response. **(C)** Responses were normalized to the amplitude of that evoked by the first application of the odor. Six cells with responses reduced by the PLC blocker were grouped separately to exemplify the effect (middle bar). **(D)** The same experiment was replicated using an intact OE expressing adenovirally induced GCaMP6f. Similar to dissociated cells, blocking PLC either did not evoke any effect (22/38 cells, **D**) or reversibly reduced the odor-evoked response (16/38 cells, **E**). **(F)** Again cells with responses reduced by the PLC blocker were grouped separately to exemplify the effect (middle bar). Asterisks denote significant difference between adjacent data sets (**p < 0.01).

In stark contrast to blocking PI3K alone, blocking PLC alone did not affect the response to the odor mixture in any of the cells tested, even in those in which blocking PI3K indicated there was inhibition to be relieved (Figures 6B,D). This was also true for 29 of a larger group of 35 dissociated ORNs not tested for their response to blockade of PI3K (Figure 7A). In 6 of the 35 ORNs, however, a significant decrease in the response to the odor mixture was measured following PLC blockade averaging 3.6 fold from 1–0.28 ± 0.15 (Figures 7B,C). Interestingly, the incidence of cells showing a decreased response following PLC blockade (6/35, ca 17%) approximates the average incidence of PI3K dependency evoked by H-100 (ca 15%). To be sure the absence of an effect of blocking PLC alone was not imposed by using dissociated ORNs, we replicated the same experiment on 38 ORNs imaged in a semi-intact preparation of live OE virally expressing GCaMP6f and obtained essentially the same result (Figures 7D–F). Together these results indicate that PLC signaling is not an obligatory component of PI-based inhibitory signaling, nor is its activation required in conjunction with odor-evoked PI3K signaling.

### Pharmacological Analysis of the Potential Contribution of PLC to the Response of ORNs to a Defined Inhibitory Odorant Pair

**Figure 8:**
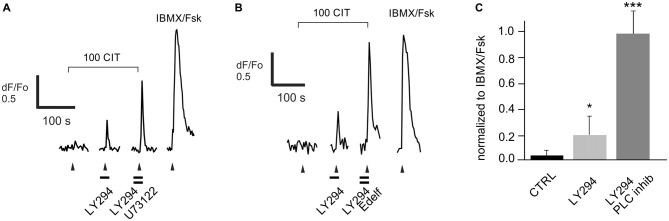
**Enhancement of the response to a known PI3K-dependent ligand citral (CIT) by both PI3K and PLC blockers.** Data were collected from five dissociated rat ORNs that were strongly excited by 50 μM octanol (OOL; not shown) and were robustly inhibited by co-application of 100 μM CIT (100 CIT). **(A)** Pre-incubation of cells with the PLC blocker U73122 (10 μM) significantly increased the PI3K blocker LY294022 (LY294)-dependent response. **(B)** A similar effect was observed in two other cells pre-incubated with a structurally distinct PLC blocker edelfosine (25 μM, Edelf). Odorant application is indicated by arrowheads. Blockers were applied at times indicated by horizontal bars. **(C)** Responses were normalized to the response activated by a mixture of IBMX and forskolin (IBMX/Fsk, 100/10 μM each). Asterisks denote significant difference between adjacent data sets (*p < 0.05; ***p < 0.001).

Previously, we identified several inhibitory odorant pairs and showed that the increase in the strength of the response following PI3K blockade reflects a PI3K-dependent increase in the agonistic strength of the “antagonist” (Ukhanov et al., 2010, 2011b). Given the possibility that the data obtained with H-100 may be confounded by the complexity of the mixture, we tested the effect of blocking PI3K and PLC on one such inhibitory odorant pair, OOL and CIT. We analyzed seven ORNs that were excited by OOL and inhibited by CIT in a PI3K-dependent manner. The response of all of these cells to CIT was increased by blocking PI3K (Figure 8) and further increased following blockade of PLC with either U73122 (Figure 8A) or edelfosine (Figure 8B). Since similar data were obtained for both PLC blockers, the results were pooled for analysis (Figure 8C). PI3K blockade increased the response to CIT (100 μM) on average 6-fold from 0.05 ± 0.02 to 0.30 ± 0.13 for PI3K blockade alone and 3-fold from 0.30 ± 0.13 to 0.88 ± 0.18 for combined PI3K and PLC blockade. This finding argues that the data obtained with H-100 were not odor-specific nor potentially confounded by the complexity of the mixture. It also suggests that the response of any one OR to pharmacological treatment is consistent; with the caveat that we cannot be certain all seven cells expressed the same OR despite their common response profile.

## Discussion

We elected to use a pharmacological approach to test whether PLC is a necessary component of a PI-based inhibitory signaling pathway in mammalian ORNs. While the question could be addressed by comparing responses of mice deficient in PLC with those of wild type animals, multiple PLC isoforms are expressed in ORNs, and even if the appropriate isoform of PLC was known, this approach would require statistical comparison of the incidence of inhibitory responses across broad samples of ORNs. Although the calcium signal manipulated pharmacologically is slow compared to the actual spike discharge of ORNs, we previously validated the correlation of these two signal types under conditions similar to those used here (Ukhanov et al., 2010).

We confirmed odor-evoked activation of PLC in ORNs using a fluorescent probe that binds specifically to PIP_2_ and translocates in response to its hydrolysis. The incidence of translocation in infected ORNs (8–13%) correlates roughly with that of PI3K-based inhibition evoked by the same odor mixture (H-100, 15%). Translocation did not occur in response to a mixture of IBMX and forskolin, suggesting that it is evoked via a cAMP-independent pathway. While these findings are consistent with PLC activation in concert with PI3K in the context of odor-evoked inhibition, they are also compatible with recently described PLC-dependent odor-evoked excitatory calcium responses (Szebenyi et al., 2014). Adding to this uncertainty, PLC probe translocation was measured in the dendritic knob, not in the olfactory cilia where transduction occurs. Thus, the odor-evoked activation of PLC we report was not necessarily occurring in the context of inhibition.

The possibility that odor-evoked activation of PLC is not related to inhibition is supported by our finding that blocking PLC alone does not enhance excitation, even in ORNs that displayed PI3K-dependent inhibition. Indeed, when an effect of blocking PLC alone was seen, it was in the opposite direction—it reduced the odor-evoked output (see below for explanation). Given that blockade of PLC alone is not sufficient to enhance odor-evoked excitation and that activation of PLC in concert with activation of PI3K is not an obligatory part of the pathway, our findings raise the question of whether the PLC effect is experimentally imposed. Both enzymes utilize a common substrate, PIP_2_ (Di Paolo and De Camilli, 2006), hinting at a logical point of interaction at which blockade of either enzyme could result in a larger pool of substrate for the other. This is particularly salient if in olfactory cilia, as in other primary cilia (Chávez et al., 2015; Garcia-Gonzalo et al., 2015), the levels of PIP_2_ are low and likely a limiting factor. Blocking PI3K could increase the pool of PIP_2_ available to PLC, resulting in a proportionally larger increase in DAG. Exogenous DAG can inhibit the heterologously-expressed olfactory CNGA2 channel (Crary et al., 2000) and also blocks the response of dissociated mammalian ORNs to odorants (Ukhanov, not shown). Increasing the pool of PIP_2_ available to PLC could either generate more DAG or increase the rate at which it is produced enough to see an effect on odor-evoked inhibition. If this is the case, however, we would expect to observe an increase in the output of ORNs resulting from PLC blockade when PI3K is blocked in a graded manner since there was clearly inhibition to be relieved, as well as an enhancing effect of blocking PLC alone in at least some ORNs, but neither of these occurred.

Conversely, blocking PLC could increase the pool of PIP_2_ available as a substrate for PI3K, resulting in a proportionally larger increase in PIP_3_. This explanation is consistent with our observations that blocking PLC increases the odor-evoked response only when PI3K is blocked and that the consequence of blocking PLC alone is a decreased response. Since the ratio of inhibitory to excitatory odorants in H-100 for any given OR differs, we assume the level of inhibition, and therefore the demand on the pool of PIP_2_, differs across cells so that the effect of blocking PLC by itself would not necessarily result in a decrease in the response of every ORN. This is consistent with our finding that blocking PLC alone failed to reduce the odor-evoked response in a few cells that displayed PI3K-dependent inhibition. Thus, there is a reasonable explanation as to how pharmacological manipulation could impose an apparent contribution of PLC to inhibitory signaling in mammalian ORNs, although more targeted experimentation is required for confirmation.

Our findings are not to suggest that PLC is without functional significance in ORNs, just that it is not necessary for the inhibitory pathway investigated here. Indeed, odor-evoked PLC activity in the transduction zone is required for there to be a pharmacologically imposed effect on inhibitory signaling. Odorants have long been known to activate PLC in isolated ciliary membrane preparations (Vogl et al., 2000), although the role of PLC in olfactory transduction has been controversial (Brunet et al., 1996; Belluscio et al., 1998; Wong et al., 2000). Recently, however, odor-activated PLC has been implicated in activity-dependent survival and stress mitigation as well as in releasing calcium from intracellular stores in mammalian ORNs (Szebenyi et al., 2014; Kim et al., 2015a). Interestingly, PI3K has also been implicated in activity-dependent survival (Kim et al., 2015b). A signaling pathway activated by the OR and involved in activation, in this case inhibition of excitation, presumably would have particular salience in promoting activity-dependent survival.

In light of the present findings, we have simplified our working model of LiSS in mammalian ORNs to emphasize PI3K as the primary mediator of PI-based inhibitory signaling. In this model, activation of an ORN by an opponent odorant pair such as OOL/CIT studied herein would involve the inhibitory odorant (CIT) activating a PI3K-dependent pathway that opposes ACIII signaling by the excitatory odorant (OOL) in a ligand selective manner. Exogeneous PIP_3_, the primary product of PI3K signaling *in vivo*, suppresses activation of the olfactory CNG channel by cAMP (Zhainazarov et al., 2004; Brady et al., 2006), indicating a downstream mechanism through which PI3K activation can mediate inhibition. Screening a panel of odorants for PI3K-dependent inhibition found that many, including some occurring together naturally in an odor object, can activate the pathway (Ukhanov et al., 2013), suggesting that this working model of olfactory LiSS is likely applicable to many mammalian ORNs.

## Author Contributions

KU performed imaging of live tissue and transfected cultured cells, immunohistochemistry and performed data analysis. KU and EC collected cells and performed RT-PCR analysis. EC designed and performed single cell RT-PCR. All authors contributed to designing the study and writing the final manuscript.

## Conflict of Interest Statement

The authors declare that the research was conducted in the absence of any commercial or financial relationships that could be construed as a potential conflict of interest.

## Footnotes

^1^http://imagej.nih.gov/ij/

^2^http://imagej.nih.gov/ij/
